# Impact of climate change on the distribution and predicted habitat suitability of two fruit bats (*Rousettus aegyptiacus* and *Epomophorus labiatus*) in Ethiopia: Implications for conservation

**DOI:** 10.1002/ece3.10481

**Published:** 2023-09-12

**Authors:** Ahmed Seid Ahmed, Afework Bekele, Mohammed Kasso, Anagaw Atickem

**Affiliations:** ^1^ Department of Biology Hawassa University Hawassa Ethiopia; ^2^ Department of Zoological Sciences Addis Ababa University Addis Ababa Ethiopia; ^3^ Department of Biology Dire Dawa University Dire Dawa Ethiopia

**Keywords:** algorithms, anthropogenic pressures, Chiroptera, climate change, ensemble model, Ethiopia, fruit bats, habitat suitability modeling

## Abstract

Fruit bats serve as crucial bioindicators, seed dispersers, pollinators, and contributors to food security within ecosystems. However, their population and distribution were threatened by climate change and anthropogenic pressures. Understanding the impacts of these pressures through mapping distribution and habitat suitability is crucial for identifying high‐priority areas and implementing effective conservation and management plans. We predicted the distribution and extent of habitat suitability for *Rousettus aegyptiacus* and *Epomophorus labiatus* under climate change scenarios using average predictions from four different algorithms to produce an ensemble model. Seasonal precipitation, population index, land‐use land cover, vegetation, and the mean temperature of the driest quarter majorly contributed to the predicted habitat suitability for both species. The current predicted sizes of suitable habitats for *R*. *aegyptiacus* and *E*. *labiatus* were varied, on average 60,271.4 and 85,176.1 km^2^, respectively. The change in species range size for *R*. *aegyptiacus* showed gains in suitable areas of 24.4% and 22.8% in 2050 and 2070, respectively. However, for *E. labiatus,* suitable areas decreased by 0.95% and 2% in 2050 and 2070, respectively. The range size change of suitable areas between 2050 and 2070 for *R*. *aegyptiacus* and *E. labiatus* shows losses of 1.5% and 1.2%, respectively. The predicted maps indicate that the midlands and highlands of southern and eastern Ethiopia harbor highly suitable areas for both species. In contrast, the areas in the northern and central highlands are fragmented. The current model findings show that climate change and anthropogenic pressures have notable impacts on the geographic ranges of two species. Moreover, the predicted suitable habitats for both species are found both within and outside of their historical ranges, which has important implications for conservation efforts. Our ensemble predictions are vital for identifying high‐priority areas for fruit bat species conservation efforts and management to mitigate climate change and anthropogenic pressures.

## Graphical abstract

The current predicted sizes of suitable habitats for *Rousettus aegyptiacus* and *Epomophorus labiatus* were varied, on average 60,271.4 and 85,176.1 km^2^, respectively. The change in species range size for *R*. *aegyptiacus* showed gains in suitable areas of 24.4% and 22.8% in 2050 and 2070, respectively. However, for *E. labiatus*, suitable areas decreased by 0.95% and 2% in 2050 and 2070, respectively. The range size change of suitable areas between 2050 and 2070 for *R*. *aegyptiacus* and *E. labiatus* shows losses of 1.5% and 1.2%, respectively.

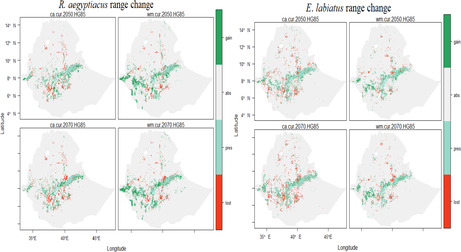

## 1 INTRODUCTION

Understanding the impact of climate change and anthropogenic pressures on wildlife distribution is essential for effective conservation planning and management (Charney et al., 2021; Jan et al., 2017; Lham et al., 2021; Urban et al., 2016; Velazco et al., 2020). The contemporary geographic ranges of mammals have declined, shifted, or been lost as a result of climate change and anthropogenic pressures (Aryal et al., 2016; Groffman et al., 2014; Jones et al., 2009; Newbold, 2018; Pinto et al., 2023; Sattar et al., 2021; Sintayehu, 2018; Thinley et al., 2021), habitat fragmentation, pollution, overexploitation, and urbanization (Barnosky et al., 2011; Carvalho et al., 2010; Marchese, 2015; Pimm et al., 2014). However, the impacts of these pressures on species distribution and the extent of suitable habitat are still poorly understood (Ali et al., 2021; Carvalho et al., 2010; Ripple et al., 2016). The effects are intensified in tropical regions as they are subject to ecological modifications (De Carvalho et al., 2017; Richardson et al., 2022; Sattar et al., 2021; Sintayehu, 2018). In response to these modifications, species migration, changes in habitat range, habitat loss, and an increased risk of extinction are further intensified (Barnosky et al., 2011; Pimm et al., 2014).

Among mammal species, bats (Order: Chiroptera) are at risk of population decline, largely due to climate change and anthropogenic pressures (Festa et al., 2023; Frick et al., 2020; Voigt & Kingston, 2016), which disrupt their natural distribution (Arumoogum et al., 2019; Boardman et al., 2020; Hughes et al., 2012; Lundy et al., 2010; Roberts et al., 2012; Stillman, 2019; Thapa et al., 2021). For instance, although one‐third of the African continent's bat species have been described, they are mostly considered endangered (15%), vulnerable (15%), and data deficient (18%) (Frick et al., 2020; IUCN, 2014; Mickleburgh et al., 2002). Many bat species in Ethiopia are classified as either data deficient or Vulnerable, such as Ethiopian *Kerivoula eriophora* (data deficient; Fahr, 2019), *Plecotus balensis* (data deficient; Benda et al., 2004; Kruskop & Lavrenchenko, 2000; Lavrenchenko et al., 2019), *Myotis scotti* (Vulnerable; Benda & Lavrenchenko, 2017). These all affect the conservation status of the species based on the impact of climate change coupled with anthropogenic pressures that need immediate solutions.

Of all bat species, fruit bats are significantly affected by climate change and anthropogenic pressures (Dey et al., 2015; Diengdoh et al., 2022; Hughes et al., 2012; Voigt & Kingston, 2016; Welbergen et al., 2008). They are vulnerable to a lack of food or resources such as fruits, nectar, and water (Del Vaglio et al., 2011; Teeling et al., 2005). Climate change is linked with seasonal changes and temperature variation, which influence the foraging behavior, food quality, and water sources of *fruit bats* (Allen et al., 2021; Arumoogum et al., 2019; Lucan et al., 2014; Pruvot et al., 2019; Weinberg et al., 2022). Anthropogenic pressures and climate change worsen habitat loss, pollution, overexploitation, urbanization, disease, roost destruction, and fragmentation (Aziz et al., 2021; Frick et al., 2020; Jan et al., 2017; Jones et al., 2009; Lok et al., 2021; Sherwin et al., 2012). These threats impact reproduction, physiology, suitable habitats, and range shifts, ultimately reducing populations (Adams, 2010; Cooper‐Bohannon et al., 2016; Moretto & Francis, 2017; Rezende et al., 2020; Schoeman et al., 2013; Sherwin et al., 2012). Consequently, climate change has forced fruit bats to migrate to new geographical ranges (Boardman et al., 2020; Eby et al., 2021; Lundy et al., 2010; Roberts et al., 2012; Stillman, 2019), which affects their survival rate and causes mortality (Dey et al., 2015; Diengdoh et al., 2022; Welbergen et al., 2008). For instance, when exposed to temperatures exceeding 42°C, over 3500 individuals of nine fruit bat species died (Dey et al., 2015; Welbergen et al., 2008). Overlooking the potential impacts of these threats on bat distribution and populations can greatly hinder conservation efforts (De Conno et al., 2018; Durant et al., 2014; Frick et al., 2020; Herkt et al., 2016; Walters et al., 2012).

Despite their affection by climate change and anthropogenic impacts, fruit bats (Pteropodidae) play critical ecological roles as bio‐indicators, pollinators, and seed dispersers (Aziz et al., 2021; De Conno et al., 2018; Fleming et al., 2009; Jones et al., 2009; Kasso & Balakrishnan, 2013; Kunz et al., 2011; Ramirez‐Francel et al., 2022; Russo et al., 2018). They are efficient pollinators due to their body size and can carry large pollen loads and seeds over long distances (Muchhala & Thomson, 2010; Ramirez‐Francel et al., 2022; Tremlett et al., 2020). On the other hand, ecosystem services improve agricultural productivity and quality of yield by providing pollinators, nutrient cycling, and natural fertilizers (Classen et al., 2014; Kasso & Balakrishnan, 2013). They also serve as reservoirs for a variety of parasites that are harmful to human health (Moratelli & Calisher, 2015).

Bats constitute 30% of mammal species in Ethiopia (Hutson & Mickleburgh, 2001; Kaipf et al., 2015; Kasso & Bekele, 2018; Wilson & Reeder, 2005), among which *R*. *aegyptiacus E. labiatus* are found (Benda et al., 2019; Happold & Happold, 2013) and they are least concern (Kaipf et al., 2015). *R*. *aegyptiacus* is mainly distributed in southern Ethiopia and the Rift Valley, while *E. labiatus* is found in the northern and Afar regions, the Rift Valley, and south‐western Ethiopia (Happold & Happold, 2013). However, Ethiopia's biodiversity is struggling to adapt to the impacts of climate change and human activities (Fashing et al., 2022; Razgour et al., 2021; Richardson et al., 2022). Specifically, research on bats in Ethiopia has been overlooked in terms of their distribution and habitat suitability (Kasso & Bekele, 2018; Kruskop et al., 2019).

As the impacts of climate change continue to intensify, species distribution models (SDMs) are increasingly utilized to determine the potential distribution ranges of species and prioritize them for conservation efforts (Charney et al., 2021; Cooper‐Bohannon et al., 2016: Hijmans & Elith, 2021; Jan et al., 2017; Lham et al., 2021; Phillips et al., 2009; Urban et al., 2016; Velazco et al., 2020). The success of many ecological models was related to the spatial characteristics of presence points and the species ranges of environmental predictors (Ancillotto et al., 2019; Bosso et al., 2022; Phillips et al., 2009). The ecological models are effective and provide clear information based on the existence of species data combined with climate change scenarios, predicting shifting and suitable habitats (Bean et al., 2012; Elith & Leathwick, 2009). We applied ensemble model predictions with a higher evaluation accuracy and increased precision as compared with a single algorithm prediction (Araujo & New, 2007; Shahnaseri et al., 2019; Thuiller et al., 2021). Since fruit bats are sensitive to anthropogenic pressures and climate change, SDMs are critical to understanding their potential distribution ranges (Diengdoh et al., 2022).

In this study, we aimed to (1) determine which environmental variables are the most important predictors for *R*. *aegyptiacus and E. labiatus*, (2) model the potentially suitable habitat distributions of the species in times of climate change, taking into account the current and future scenarios (2050 and 2070), and (3) assess the spatiotemporal species range changes for conservation implications. We hypothesize that climate change influences the distribution of suitable habitats for the target species. The predictions of this ensemble model can be used to identify areas that should be given high priority for fruit bat conservation and ecosystem improvements, as well as to initiate further research on the topic.

## 2 MATERIALS AND METHODS

### 2.1 Species occurrence

The present study was conducted in Ethiopia which is located within the tropics (3° and 15° N latitude and 33° and 48° longitude, respectively; Figure S1). It has an elevation range from 125 m below sea level in the Danakil Depression to 4533 m above sea level in the Simien Mountains (IBC, 2005).

We compiled all occurrence points for the species found exclusively in Ethiopia using a combination of a personal survey and secondary data sources such as literature (Benda et al., 2019; Kaipf et al., 2015) (*n* = 6 for *R*. *aegyptiacus* and 4 for *E. labiatus*), and from Global Biodiversity Information Facility (GBIF) (*n* = 7 for *R*. *aegyptiacus* and *n* = 10 for *E. labiatus*) (GBIF.org, 2022a, 2022b). The surveys were conducted from June 2020 to December 2022 at representative roosting sites such as in eastern Ethiopia (Dire Dawa in Tomi Farm, the water tower, and Enkuftu Cave), western Hararghe (Dindin Forest, Kuni Muktar Forest, Anchare Cave, Holka Chire, Aynage Cave, and Bero Forests and Caves), Babile elephant sanctuary and buffer zones, south‐west Ethiopia (Omo National Park and Hana Area), the Rift Valley (Awash National Park area), and the central (Guassa community conservation areas), Abijatta‐Shalla National Park, and *Menagesha* Suba Forest Park. We trap bat using five mist nets with 12 m long and 3 m wide and 10 Ecotone telescope mist‐net poles. Mist nets were stretched in and around roosting sites, forest edges, caves, and their entrances. Mist netting was conducted between 7:00 p.m. and 9:00 p.m. Capturing and handling specimens conform to the principles and guidelines of Mammals of Africa, Vol. IV (Happold & Happold, 2013).

Before fitting the model, occurrence points were rarified and aligned to a 1 km^2^ raster grid resolution of bioclimatic variables using SDMtoolbox version 2.5 in ArcGIS v10.7. A total of 86 and 95 occurrence points were used after duplication removal for *R*. *aegyptiacus* and *E. labiatus*, respectively, to model the species distribution. In addition, we generated ten thousand pseudo‐absence points at random in 20‐kilometer buffer zones around the presence points and rarified them by keeping 1 km^2^ spatial resolution. We generated the sample with data (SWD) by combining and rarifying occurrences and pseudo‐absences represented by 0 and 1 in the CSV files using ArcGIS version 10.7 and using SDM tools, pseudo‐absence points also have the greatest impact on prediction powers to identify the predictor variable (Barbet‐Massin et al., 2012).

### 2.2 Environmental variables

We considered 29 environmental variables, including 19 bioclimatic variables, land cover, human population index, altitude, aspect, ecoregion, water area, waterlines, vegetation, slope, and slope standard deviation, for building a model of fruit bat species. The bioclimatic variables were extracted from WorldClim 2.1 at a spatial scale resolution of 30 arc seconds (~1 km^2^; Fick & Hijmans, 2017). The topographic attributes were extracted from the Shuttle Radar Topography Mission digital elevation model (SRTM DEM; Jarvis et al., 2008), ecoregion from Olson et al.  (2001), vegetation from (http://landscapeportal.org/layers/geonode:veg_ethiopia), and human population (https://data.humdata.org/organization). The water area and water lines shapefiles were retrieved from (https://www.diva‐gis.org/datadown), and the distance to the water area and water lines was calculated using the Euclidian distance by keeping the climatic variable resolution. The topographic attributes, population, and vegetation were resampled to fit a spatially resolved bioclimatic variable using ArcGIS 10.7. We used all the selected variables for current (baseline) predictions (1970–2000) as well as future projections for 2050 (from 2040 to 2060) and 2070 (from 2061 to 2080). The HadGEM2‐ES global circulation model (GCM) developed by the UK Met Office Hadley Centre was applied for future modeling, which included two shared socioeconomic pathways (SSPs) for 2050 and 2070: the intermediate (SSP 4.5) and the worst (SSP 8.5).

We performed a multicollinearity test using a variance inflation factor (VIF) to avoid highly correlated predictor variables. All the variables were extracted using the retained occurrence points and randomly generated 10,000 points to compute the Pearson correlation among the variables. Then, using the USDM package (Naimi, 2017) in R version 4.1.2, the variables with VIF ≤5 and correlation coefficient (*r*) ≤ |.8| were retained for the final model building (Naimi et al., 2014). Finally, we used 14 environmental variables for the modeling of suitable habitats for *R*. *aegyptiacus* and *E. labiatus* (Table S1).

### 2.3 Model fitting and evaluation

We selected the species distribution algorithms based on their characteristics, their suitability for the available data, and their intended application (Aertsen et al., 2010, 2011; Kampichler et al., 2010; Li & Wang, 2013). We used the ensemble model by averaging four different algorithms, namely: Generalized linear model (GLM), Generalized boosted model (GBM), Random Forest (RF), and Maximum entropy (MaxEnt; Thuiller et al., 2009, 2021). These models have higher predictive powers (Kaboodvandpour et al., 2021) and are widely used in academic research and species conservation (Bosso et al., 2022). These models were classified as regression‐based models such as GLM and machine learning models such as GBM, RF, and MaxEnt (Fitzgibbon et al., 2022; Hallgren et al., 2019; Halvorsen et al., 2015; Hao et al., 2019; Hijmans & Elith, 2021; Loh, 2011). We applied the ensemble and biomod2 R software packages (Thuiller et al., 2021).

We applied the 10‐fold cross‐validations run with three replications, 2500 and 500 number of trees for the GBM and RF algorithms, respectively (Elith et al., 2008; Thuiller et al., 2021). Under GLM functions, the formula is defined as a quadratic setting (Li et al., 2021, 2022; Thuiller et al., 2021). For MaxEnt, we produced 10,000 background points at random and 5000 iterations, we used default parameters for each method while taking into consideration the features of the modeling framework (Barbet‐Massin et al., 2012; Thuiller et al., 2021). We have evaluated the results of the default and various setting parameters before building the model, like background points, iterations, learning rate, features, and number of trees. Dormann et al. (2018) mentioned that the ensemble model predictions were created using different algorithms, and each prediction was averaged to enhance prediction performance and minimize uncertainty. We calibrated the model validations by splitting the data into 3:1 ratios (70 used for model calibration and 30% to evaluate the current predictive performances; Phillips et al., 2006).

The accuracy of the model prediction was evaluated using a receiver operating characteristic curve (ROC), true skill statistic (TSS), and Kappa (Cohen's Kappa; Allouche et al., 2006; Assefa et al., 2022; Breiner et al., 2018; Eskildsen et al., 2013; Shabani et al., 2018). AUC values range from 0 to 1, with values >0.7 indicating useful validation (Georges & Thuiller, 2013; Mandrekar, 2010). Kappa statistic values greater than 0.55 indicate good evaluation performances (Duan et al., 2014; Monserud & Leemans, 1992). The models with a true skill statistic (TSS) value greater than 0.7 were selected as a weighted set to form an ensemble model. This is sufficient for internal evaluation of the predictions (Ahmad et al., 2020). Evaluating the performance of the baseline current prediction (1970–2000) was done using three threshold cutoff criteria, including the mean of the probabilities, committee averaging (ca), and the probability weighting mean (wm; Hao et al., 2019; Thuiller et al., 2021). The probabilities calculated from several models are averaged in committee averaging. The probability weighting mean is determined by dividing the total of the weights by the sum of the projected probabilities divided by the corresponding weight (Araujo & New, 2007; Thuiller et al., 2021). We used two threshold criteria for the current and future predictions, and determined their intersections based on the score graph TSS versus ROC metrics: committee averaging and weighted mean criteria (Figure S3). To obtain the result of the ensemble model, we multiplied the value of each grid in the single model by the weight of the corresponding model and then summed them. The values at the threshold were considered suitable areas in the species' final prediction maps. We predicted the species' range size change using the current and future (Curr_2050 and Curr_2070) intersections by using two thresholds that produce better results under the two shared socioeconomic pathways, SSP4.5 (intermediate) and SSP8.5 (worst). The predicted areas of unsuitable, suitable, loss, gain, remaining suitable, species range change, and future range were calculated using the Biomod2 package (Thuiller et al., 2021) in R version 4.1.2.

## 3 RESULTS

### 3.1 Variables contributions that predict habitat suitability of *R*. *aegyptiacus* and *E. labiatus* under climate change

On average, the result shows that the most influential factors determining the distribution and habitat suitability of *R*. *aegypticus* are seasonal precipitation (23.5%), population index (22.7%), land use and land cover (20%), vegetation (16.9%), and ecoregions (14.3). Whereas in *E. labiatus*, the most influential covariates are population index (34.2), seasonal precipitation (26.7%), *land* use land covers (21.4%), vegetation (18%), the mean temperature of the driest quarter (14.3%), temperature annual range (13.1%), and ecoregion (12.3%) (Table S2).

### 3.2 Current predicted suitable habitats

Our averaged model performance shows high‐evaluation metrics values of AUC, TSS, and Kappa, which were very good for discriminating suitable habitats from unsuitable areas (Table 1). We used committee averaging and probability of weighted mean values from the ensemble models' predictions for *R*. *aegypticus* and *E. labiatus* (Table 1).

**TABLE 1:** The probability of committee averaging (ca) and the probability of weighted mean (wm) metrics predict the distribution and habitat suitability of *R*. *aegypticus* and *E. labiatus* by utilizing average predictions from different algorithms.

Ensemble models' evaluation methods				
Species	Metrics	ca	wm	Average
*R. aegypticus*	AUC	.99	.99	.99
	TSS	.93	.95	.94
	KAPPA	.96	.65	.75
*E. labiatus*	AUC	.98	.98	.98
	TSS	.89	.88	.87
	KAPPA	.86	.65	.72

**FIGURE 1:**
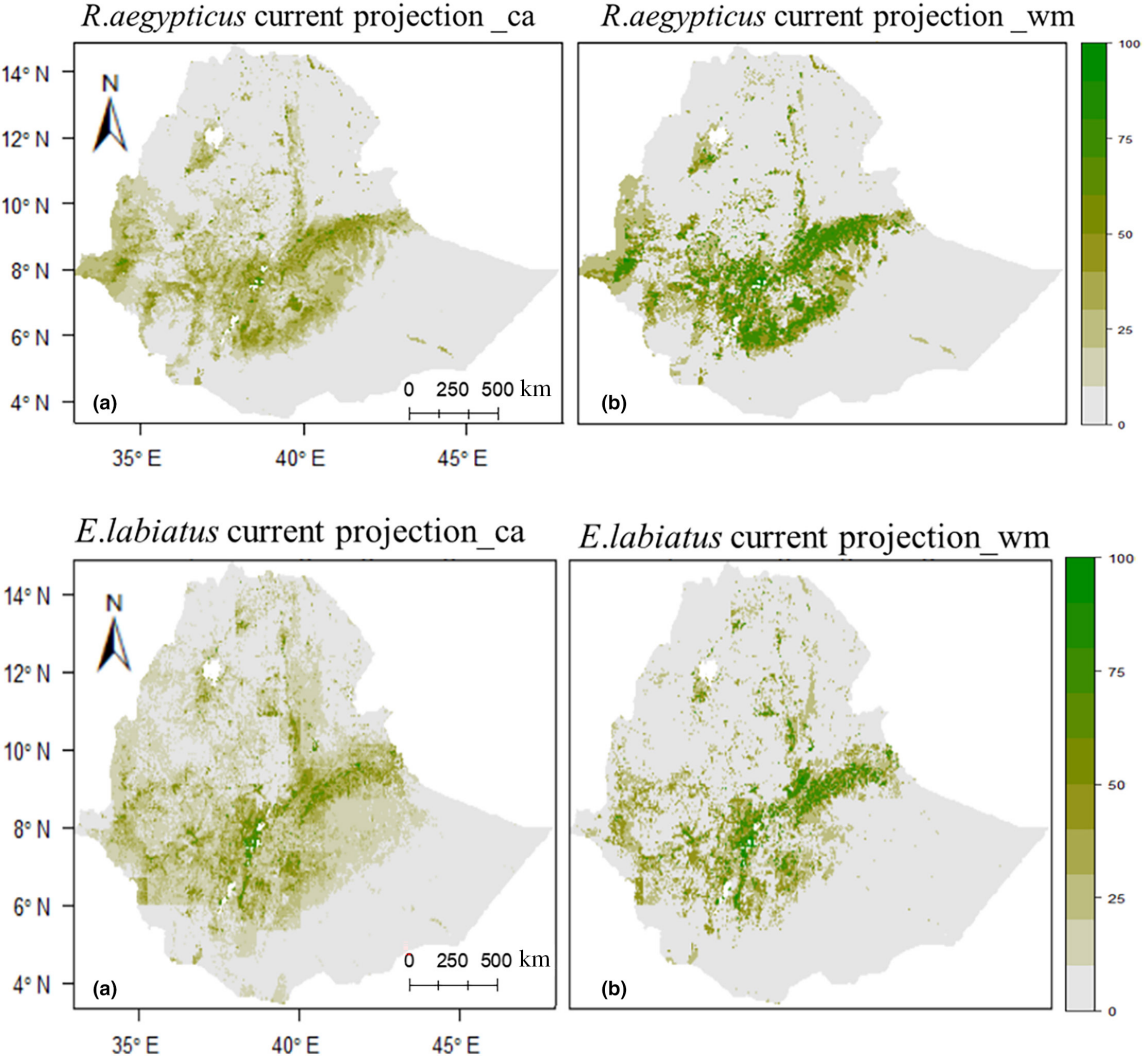
The ensemble current distribution and habitat suitability maps of *Rousettus aegyptiacus* and *Epomophorus labiatus* were produced using the probabilities of committee averaging (ca) predictions (a) and the probabilities of weighted mean (wm) predictions (b), respectively. The color scale indicates the percent habitat suitability indexes.

The current prediction shows that *R*. *aegypticus* and *E. labiatus* had 60,271.4 and 85,176.1 km^2^ of suitable habitat, respectively. The average predicted maps show that *R*. *aegypticus* and *E. labiatus* have high‐suitable habitat index values and are located in southern and eastern Ethiopia's highlands (Figure 1). Furthermore, fragmented suitable areas were predicted in central Ethiopia and northern highlands, along the borders of Amhara, Afar, and Tigray, as well as around Lake Tana (Figure 1).

### 3.3 Future prediction

**FIGURE 2:**
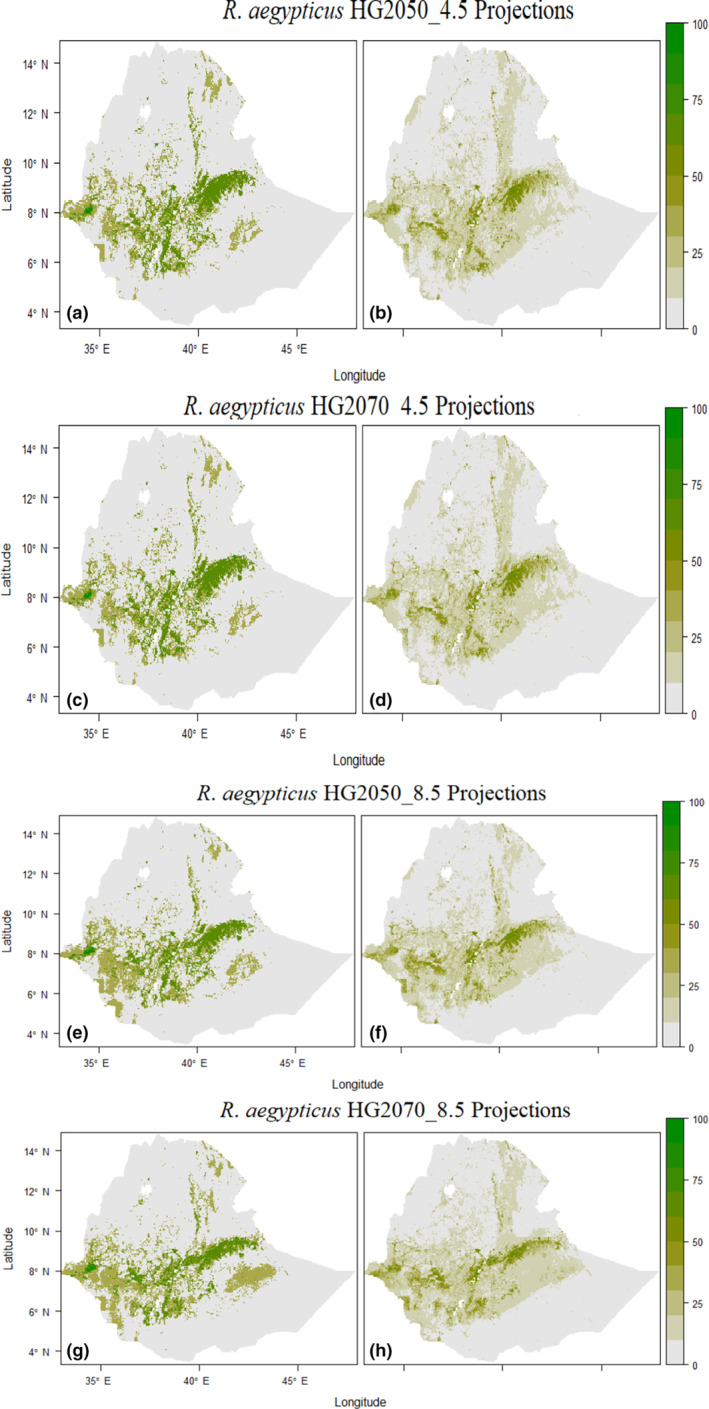
Distribution and habitat suitability map of *Rousettus aegyptiacus* projected for the future (2050 and 2070) periods ensemble using the committee averaging (ca) across predictions (a, c, e, g) and the probability of weighted mean (wm) (b, d, f, h), respectively. The color scale indicates the percent habitat suitability indexes.

**FIGURE 3:**
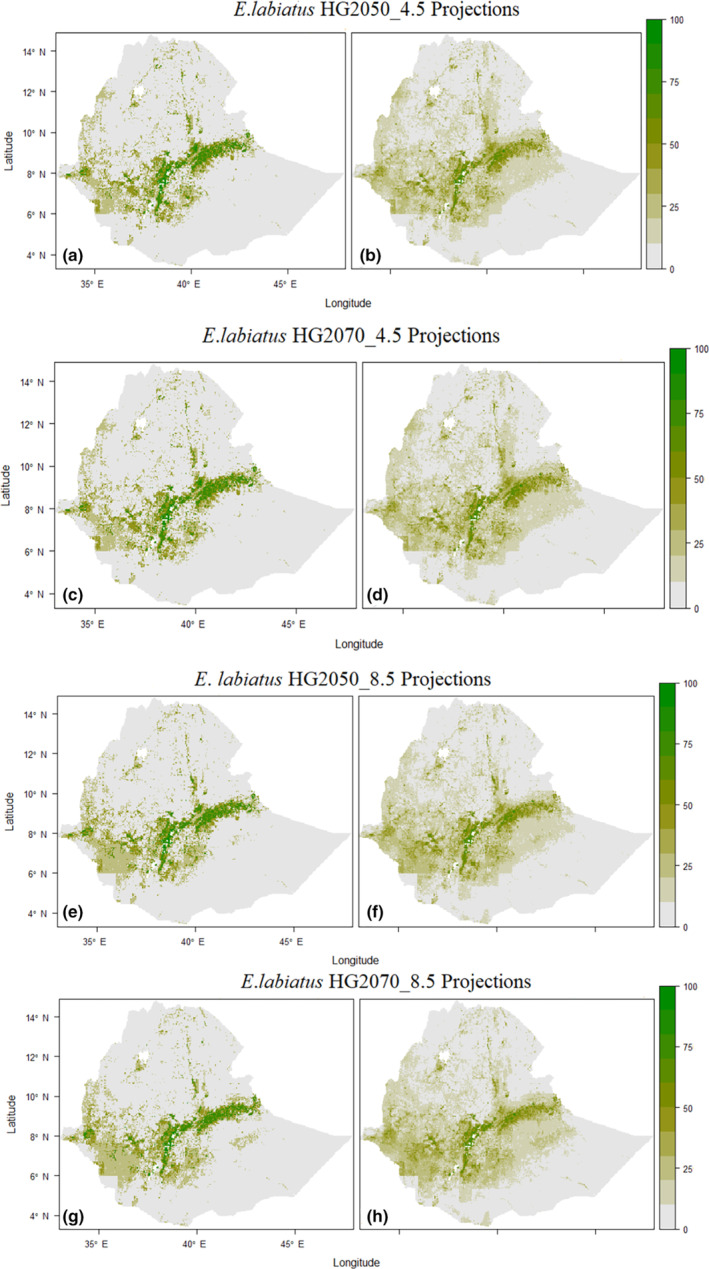
Distribution and habitat suitability map of *Epomophorus labiatus* projected for the future (2050 and 2070) periods ensemble using the committee averaging (ca) across predictions (a, c, e, g) and the probability of weighted mean (wm) (b, d, f, h), respectively. The color scale indicates the percent habitat suitability indexes.

**FIGURE 4:**
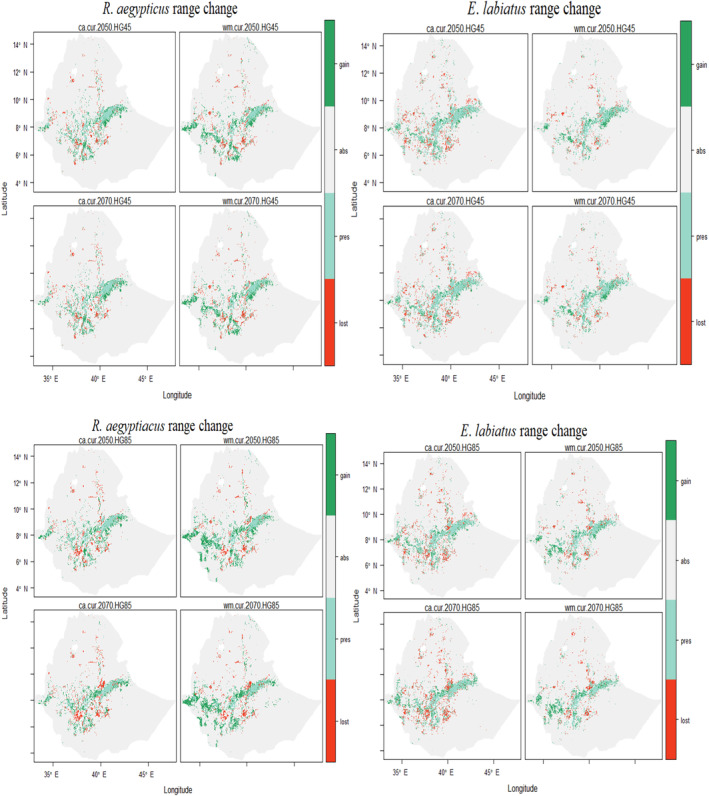
The projected maps show the area change by the overlap of the current and future scenarios for *Rousettus aegyptiacus* and *Epomophorus labiatus* (curr_2050 HG45, curr_2050 HG85, curr_2070 HG45, and curr_2070 HG85) produced at two threshold levels of the models (ca: the committee averaging across predictions and wm: the probability of weighted mean, respectively). The color scale indicates the percent habitat suitability indexes.

Compared with the current tbaseline predicted range, *R*. *aegypticus* is predicted to have a notable gain in future scenarios (Figure 2, Figure S4). However, *E. labiatus* is predicted to experience a reduction in future range size (Figure 3, Figure S4, Table 2). The averaged results show the future projections for suitable habitat for *R*. *aegypticus* in 2050 and 2070 were predicted to be 77,412.7 and 76,249.6 km^2^, respectively (Figure 4, Table 2). The average future projections of suitable habitat for *E. labiatus* were 84,226.2 and 83,253.8 km^2^ in 2050 and 2070, respectively (Figure 4, Table 2).

**TABLE 2:** The ensemble model results consider the average of the committee averaging across predictions (ca) and the probability of weighted mean (wm) for the species range size change in each scenario for *R*. *aegypticus* and *E. labiatus.*

Scenarios	Species	Loss, km^2^	Unsuitable, km^2^	Remain suitable, km^2^	Gain, km^2^	% loss	% gain	Species range size change (%)	Current range size, km^2^	Future range size, km^2^
2050	*R. aegyptiacus*	17,601.835	1,041,977.72	42,669.545	34,743.14	25.155	49.579	24.424	60,271.38	77,412.728
	*E. labiatus*	21,193.41	1,031,499.48	63,982.71	20,243.54	21.371	20.425	0.946	85,176.12	84,226.25
2070	*R*. *aegyptiacus*	19,224.225	1,041,518.48	41,047.155	35,202.38	27.391	50.224	22.833	60,271.38	76,249.578
	*E. labiatus*	23,218.065	1,030,447.27	61958.055	21,295.75	23.478	21.5	1.978	85,176.12	83,253.805

Our projected maps mainly lined up with the predicted suitable areas in the southern and eastern Ethiopian highlands, and the southern Rift Valley areas were highly suitable for *R. aegypticus* and *E. labiatus* (Figure 4). All the projected maps indicate that there are fragmented areas in the northern, western, and central highlands, as well as along the borders between the Afar and Amhara regions. The projected changes in range size for both species are characterized by visible fluctuations in the predicted suitable habitats, with some areas gaining and others losing suitable habitat (Figure 4).

## 4 DISCUSSION

Our study shows that good performance across all averaged evaluation metrics leads to greater power to identify the distribution and suitable habitat priority areas. The current predicted AUC values were greater than those of *R*. *aegyptiacus* (Arumoogum et al., 2019; Bilgin & Rebelo, 2012; Kafash et al., 2022; Wordley et al., 2015). It was also comparable to the prediction performance of *R*. *aegyptiacus* (Cooper‐Bohannon et al., 2016). Furthermore, TSS results also show better performances than *R*. *aegyptiacus* (Arumoogum et al., 2019).

In the present study, seasonal precipitation was the main climatic variable affecting the predicted habitat suitability of *R*. *aegyptiacus*, followed by population index, land cover, and vegetation. On the other hand, seasonal precipitation had the greatest impact on the predicted habitat suitability *of E. labiatus*, next to the population index. This result is consistent with the findings that seasonal precipitation has a significant impact on the distribution and suitability of bats (Bandara et al., 2022; Bilgin & Rebelo, 2012). In addition, it impacts the foraging and social structures of bats, potentially delaying reproduction and increasing the risk of their survival (Adams, 2010; Frick et al., 2010; Luo et al., 2020; Mello et al., 2009; Richter & Cumming, 2008; Sherwin et al., 2012; Weinberg et al., 2022). Recent studies show that the foraging behavior, food quality, and water sources of *R*. *aegyptiacus* are influenced by seasonal changes in temperature, precipitation in the driest quarter, and annual temperature (Arumoogum et al., 2019; Bilgin & Rebelo, 2012; Lucan et al., 2014). Furthermore, precipitation had a great impact on the metabolic rates of fruit bats (Arumoogum et al., 2019) and their thermoregulatory systems (Downs et al., 2012). The seasonality of precipitation on a global scale can have significant impacts on wildlife behavior, foraging activity patterns, and trophic interaction (Sattar et al., 2021). When coupled with temperature, it might strongly affect food availability, hibernation, physiology, and reproduction in fruit bats. These, in turn, can also affect the growth and distribution of flowering plants, including the timing and duration of flowering as well as the quality and quantity of fruit produced.

Anthropogenic pressures coupled with climate change significantly impact the geographic range of fruit bats, such as *R*. *aegypticus* (Cooper‐Bohannon et al., 2016; Moretto & Francis, 2017; Rezende et al., 2020; Schoeman et al., 2013). These factors have caused changes in precipitation, temperature, droughts, and wildfires that have impacted bats habitat, to which the fruit bats have responded rapidly (Allen et al., 2021; Pruvot et al., 2019; Weinberg et al., 2022). Our results indicate that population index and land use and land cover changes have a great impact on the predicted suitable habitat for the targeted species. Land use and land cover change can significantly impact the distribution and range shifts of fruit bats by affecting food availability and roosting (Arumoogum et al., 2019; Hughes et al., 2012; Taheri et al., 2021). Intensive agricultural cultivation and concentrated human settlements have a significant impact on the distribution and colonization of *R*. *aegyptiacus* (Hulva et al., 2012; Kafash et al., 2022). These impacts may be linked to the availability of prey, climate, and roosting sites (Egert‐Berg et al., 2021; Hulva et al., 2012; Williams‐Guillén et al., 2008; Weinberg et al., 2022). In addition, intensive farming has been identified as a contributing factor to conflicts between humans and fruit bats as possible agents of zoonotic diseases (Fill et al., 2022; Majumdar et al., 2016; Ramanantsalama et al., 2022; Roberts et al., 2016). Despite the challenges, fruit bats can often be found in cultivated and urban areas, specifically targeting these areas (Majumdar et al., 2016; Roberts et al., 2016).

Vegetation is also an important predictor that influences habitat suitability for fruit bat species. Dry and moist deciduous forests, native plants, and angiosperm plants, as well as caves, provide suitable roosting habitat for *R*. *aegyptiacus* and *E. labiatus* (Happold & Happold, 2013; Majumdar et al., 2016; Mphethe et al., 2023; Roberts et al., 2016). Different ecoregions, landscapes, and vegetation all have an impact on fruit bat dispersal patterns because they offer food and roosting protection (Frick et al., 2020; Hughes et al., 2012; Kafash et al., 2022). Forest‐dependent fruit bats rely on fruit plants for food and roosting throughout the year (Egert‐Berg et al., 2021; Frick et al., 2020; Kafash et al., 2022; Lucan et al., 2014; Mphethe et al., 2023). However, they are threatened by habitat loss, distributional shifts, and population decline due to changes in forest structures (Fill et al., 2022; Frick et al., 2020; Hughes et al., 2012; Kafash et al., 2022; Moretto & Francis, 2017).

The predicted maps show that both bat species had highly suitable areas concentrated in the southern and central‐eastern regions of Ethiopia's highlands. However, there were fragmented, suitable areas in the western and northern parts of the highlands. The predicted distribution range of *R*. *aegyptiacus* and *E. labiatus* is mostly consistent with the previous reports (Benda et al., 2019; IUCN, 2016; Happold & Happold, 2013). The projected change in species range size for *R*. *aegyptiacus* showed a gain in suitable habitats by 24.4% and 22.8% in 2050 and 2070, respectively. However, for *E. labiatus*, the species range size change indicates a reduction of 0.95% and 2% in 2050 and 2070, respectively. It is important to consider that different species may react differently to predicted changes, which has significant implications for conservation efforts (Singh et al., 2018). In addition, the range size change between 2050 and 2070 for *R*. *aegyptiacus* and *E. labiatus* shows a reduction of 1.5% and 1.2%, respectively. The projected range map showed that climate change is likely to have a notable impact on the distribution and suitability of habitats for these two species. The maps indicated that the predicted ranges of suitable habitats for the species were lost and labeled with red (Figure 4), indicating a loss of suitable habitat. The study also examined variations in the projections of range change, as well as gains and losses in expected suitable areas, to further identify the influence of climate change (Jones et al., 2013). The impacts of climate change were defined by bat species range changes in terms of gains, losses, and shifting ranges (Arumoogum et al., 2019; Diengdoh et al., 2022; Hughes et al., 2012; Jones et al., 2013; Rebelo et al., 2010; Thapa et al., 2021). Human activities and climate change are posing a threat to bat distribution and ecosystem services, making bats more vulnerable to changes in their range (Knight, 2022; Razgour et al., 2021).

Our study predicted suitable habitats for both species within and outside of their historical distribution ranges, with *E. labiatus* showing particularly notable results (Figure S2). Similar studies on fruit bats, such as *Pteropus poliocephalus*, *Pteropus alecto* identified predicted suitable habitat ranges outside of their expected ranges (Van Der Ree et al., 2006; Diengdoh et al., 2022). Shortages of food, destruction of roosting sites, and environmental pressures might be factors that influence the geographic ranges of fruit bats. The geographical ranges of fruit bats have changed over time due to various factors, including responses to acute food shortages and climate change. This can lead to the colonization of previously uninhabited regions (Diengdoh et al., 2022; Eby et al., 2021; Festa et al., 2023; Hulva et al., 2012; Kafash et al., 2022). Fruit bats, including *R*. *aegyptiacus* and *E. labiatus*, are known for their ability to travel long distances and adapt to changing environmental conditions. This allows them to explore new geographical landscapes in search of better food resources and roosting sites (Benda et al., 2012; Egert‐Berg et al., 2021; Weinberg et al., 2022). These species may respond through range shifting, migration, and declines in species distribution (Sherwin et al., 2012), which increase vulnerability and mortality (Downs et al., 2012). Through mapping and predicting a species' distribution and habitat suitability, it is possible to identify species priorities for future conservation planning (Charney et al., 2021; Erfanian et al., 2021; Velazco et al., 2020).

The present prediction of the target species distribution and habitat suitability provides a valuable baseline for future conservation planning by identifying priorities. Conservation recommendations based on predictions of changes in habitat suitability must be approached with caution due to potential variations in how different species may respond (Kufa et al., 2022; Singh et al., 2018). Conservation bats are important due to their crucial role in maintaining biodiversity and providing essential ecosystem services such as pest management in agriculture, pollinators, and malaria control (Fill et al., 2022; Riccucci & Lanza, 2014). Effective conservation and rehabilitation of the programs are dependent on climate trends and the distribution ranges of species (Préau et al., 2020). Understanding bat distribution ranges and environmental impacts using ensemble prediction is important for effective conservation strategies. Although we used averaged predictions to reduce uncertainty, the use of a single global circulation model is a limitation of our study. Prioritizing future research on how fruit bats respond to habitat fragmentation and landscape structures, as well as understanding their ecological values, can inform the development of fruit bat conservation measures in the future.

## 5 CONCLUSION

The present study predicted the potential distribution and suitable habitat for *R*. *aegypticus and E. labiatus under* current and future scenarios. We identified the dominant environmental variables that affect the predicted distribution and suitable habitat of the species. Understanding the current and future distribution of suitable areas for the fruit bat is crucial for the conservation and management of volant fauna. Our study highlights the vulnerability of fruit bats to climate change, with *R*. *aegyptiacus* indicating a gain in suitable areas and *E. labiatus* showing a reduction. The study suggests suitable habitats for both species exist within and outside of their historical ranges, providing valuable insights for conservation strategies. This highlights the need to consider suitable habitats beyond reported ranges when developing conservation strategies to ensure the long‐term health and sustainability of ecosystems. The positive ecological role of fruit bats in pollination and seed dissemination, as well as their associated concerns about human–wildlife conflict, have significant ecological and societal consequences. Our results provide valuable information for establishing a baseline, identifying priority areas for restoration, informing conservation efforts, and planning for future management of extant fruit bat populations. By taking action to protect targeted species and their habitats, we can help ensure the continued health and sustainability of ecosystems while also supporting the well‐being of human communities that rely on the ecosystem services provided by these fascinating mammals.

## AUTHOR CONTRIBUTIONS

**Ahmed Seid Ahmed:** Conceptualization (lead); data curation (lead); formal analysis (lead); investigation (lead); methodology (lead); project administration (lead); validation (lead); writing – original draft (lead); writing – review and editing (lead). **Afework Bekele:** Supervision (equal); validation (equal); writing – review and editing (equal). **Mohammed Kasso:** Writing – review and editing (supporting). **Anagaw Atickem:** Funding acquisition (lead); validation (lead); writing – review and editing (lead).

## CONFLICT OF INTEREST STATEMENT

The authors declare that there is no conflict of interest.

## Supporting information


Data S1

## Data Availability

Data are available in the supplementary material and in the hands of Correspondence authors.
