# Supplementary material for: Impact of climate change on the distribution and predicted habitat suitability of two fruit bats (Rousettus aegyptiacus and Epomophorus labiatus) in Ethiopia: Implications for conservation
Source: Ecol Evol. 2023 Sep 12;13(9):e10481. doi: 10.1002/ece3.10481 (PMC10497737; doi:10.1002/ece3.10481)
Supplement: Supplementary file 1 — Data S1 [file ECE3-13-e10481-s001.docx]

**Supplementary materials**

**Title: Impacts of climate change on distribution and the predicted habitat suitability of two fruit bats (*Rousettus aegyptiacus* and *Epomophorus labiatus*) in Ethiopia: implications for conservation**

Ahmed Seid Ahmed^a, b,^ *, Afework Bekele^b^, Mohammed Kasso^c^_,_ and Anagaw Atickem^b^

^a^ *Department of Biology, Hawassa University, P. O. Box 05, Hawassa, Ethiopia*, Orcid ID: 0000-0001-9024-4532

^b^ *Department of Zoological Sciences, Addis Ababa University, P.O. Box. 1176, Addis Ababa, Ethiopia, email address:* [anagawam@gmail.com](mailto:anagawam@gmail.com)*,* Orcid ID: 0000-0002-2397-6108

^b^ *Department of Zoological Sciences, Addis Ababa University, P.O. Box. 1176, Addis Ababa, Ethiopia, email-address:* [afeworksimegn@gmail.com](mailto:afeworksimegn@gmail.com)

^c^ *Department of Biology, Dre Dawa University,* P**.**O. Box:1362*, Dire Dawa, Ethiopia, email address:* muhesofi@yahoo.com*,* Orcid ID*:* 0000-0002-3863-4848

^*^Correspondence: [ahmedse4378@gmail.com](mailto:ahmedse4378@gmail.com)


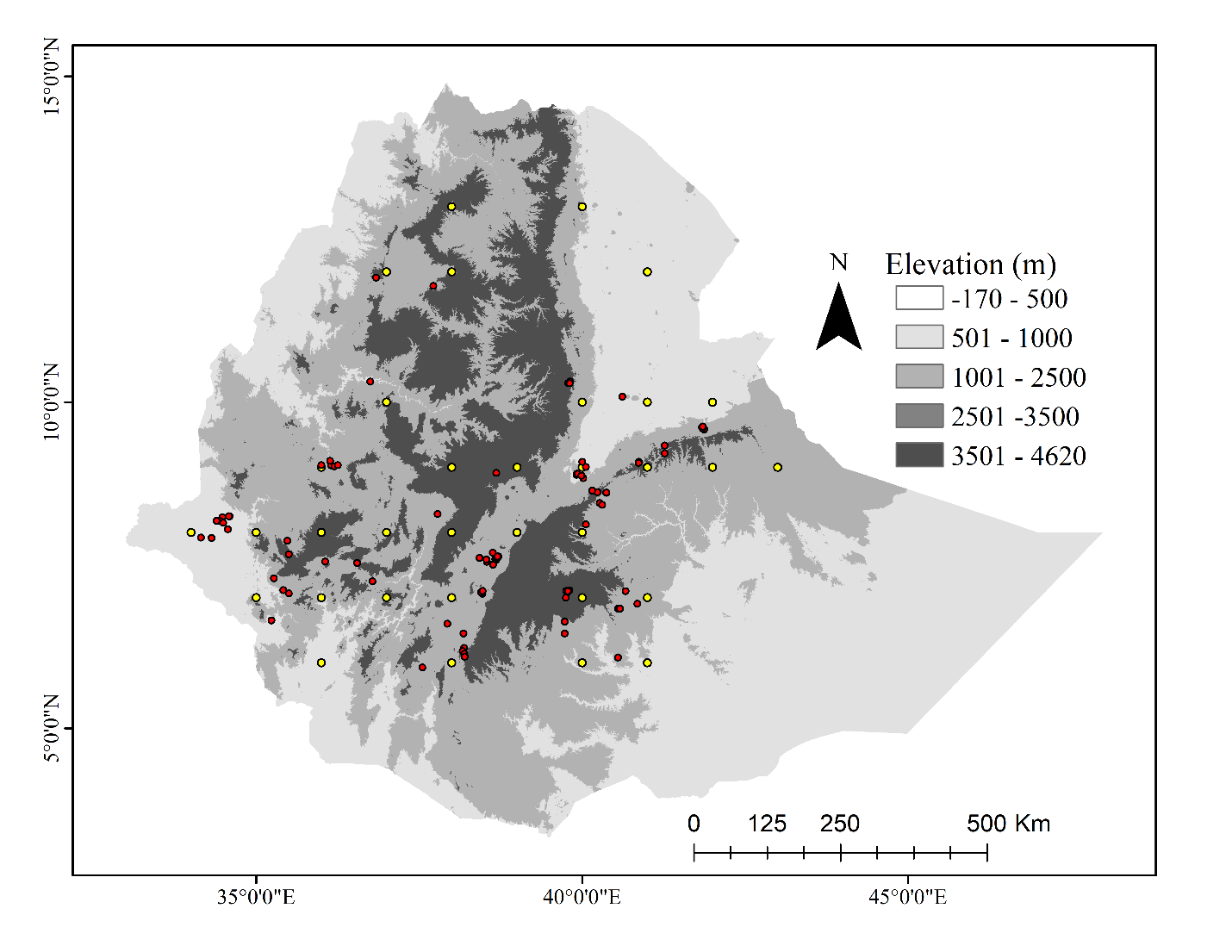


Figure. S1 The map of Ethiopia's and occurrence points of *R*. *aegyptiacus* (red dots) and *E*. *labiatus* (yellow dots).


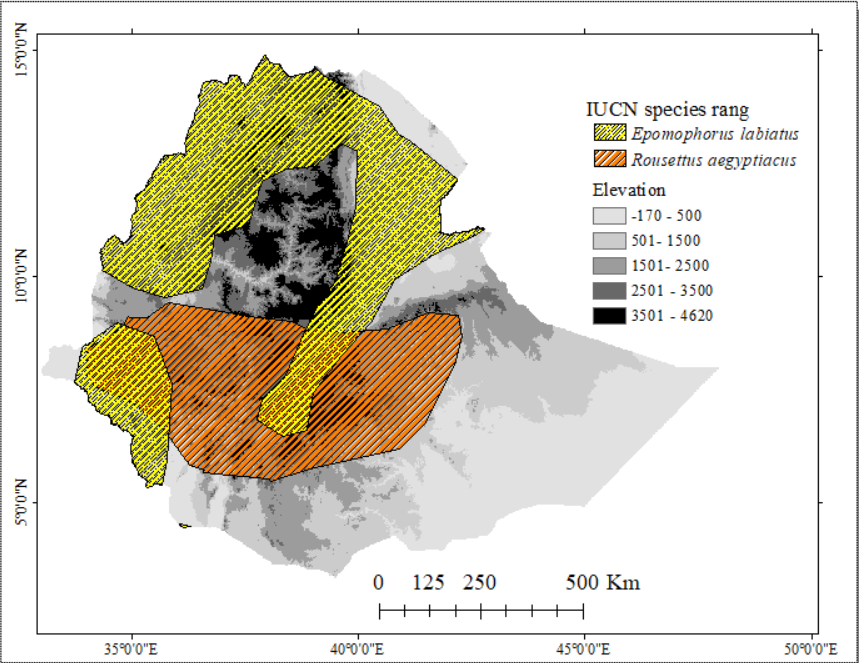


Figure. S2 IUCN reports of the two fruit bats ***Rousettus*** aegyptiacus ***(orange) and*** Epomophorus labiatus **(**yellow**)**

**Table S1** Environmental predictor variables retained for modeling the habitat of bats and their codes, units of measurement, and data sets

| **Category** | **Variables** | **Code/unit** | **Source** |
| --- | --- | --- | --- |
| **Bio climate** | Temperature Annual Range | Bio7/^0^C | (<https://worldclim.org/data/worldclim21.html>) |
|  | Mean Temperature of Driest | Bio9/^0^C |  |
|  | Precipitation of driest month | Bio14/mm |  |
|  | Precipitation Seasonality | Bio15/mm |  |
|  | Precipitation of warmest quarter | Bio18/mm |  |
|  | Precipitation of coldest quarter | Bio19/mm |  |
| **Anthropogenic** | Land use land cover | LuLc/unitless | https://cds.climate.copernicus.eu/ |
|  | Human population density | PopIndex/unitless | <https://data.humdata.org/organization> |
| **Inland water** | Distance to water areas | WA/m | <https://www.diva-gis.org/datadown> |
|  | Distance to water lines | WL/m |  |
| **Topographic** | Ecoregion | Ecoreg/ unitless | <https://lpdaacsvc.cr.usgs.gov/appeears> |
|  | Slope | Slope/ unitless |  |
|  | Aspect |  |  |
|  | Vegetation | Vegete/ unitless | <http://landscapeportal.org/layers/geonode:veg_ethiopia> |


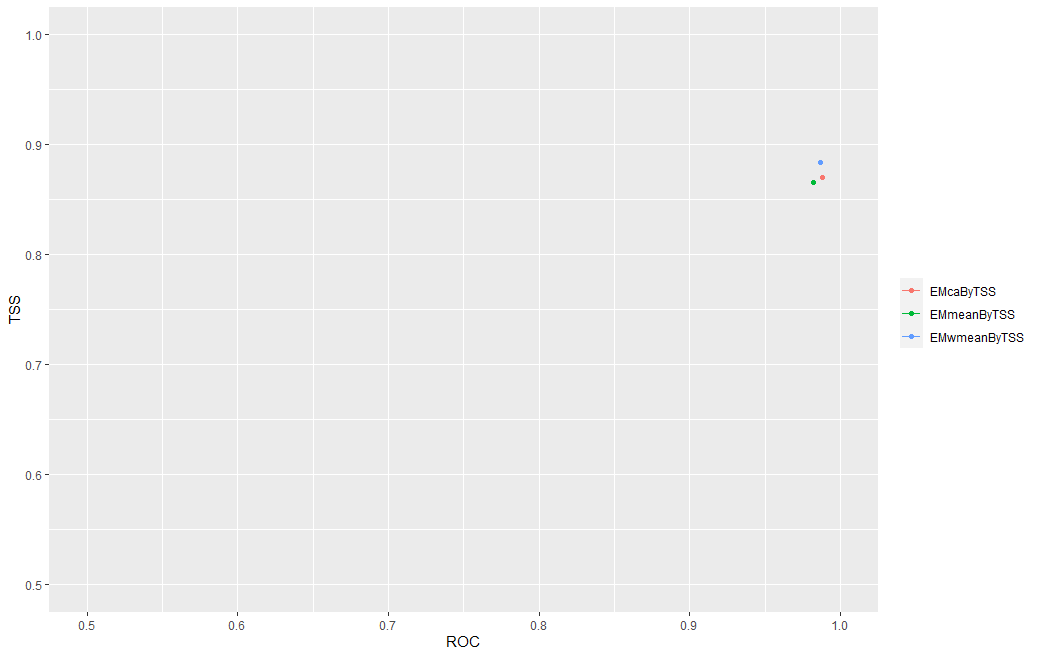

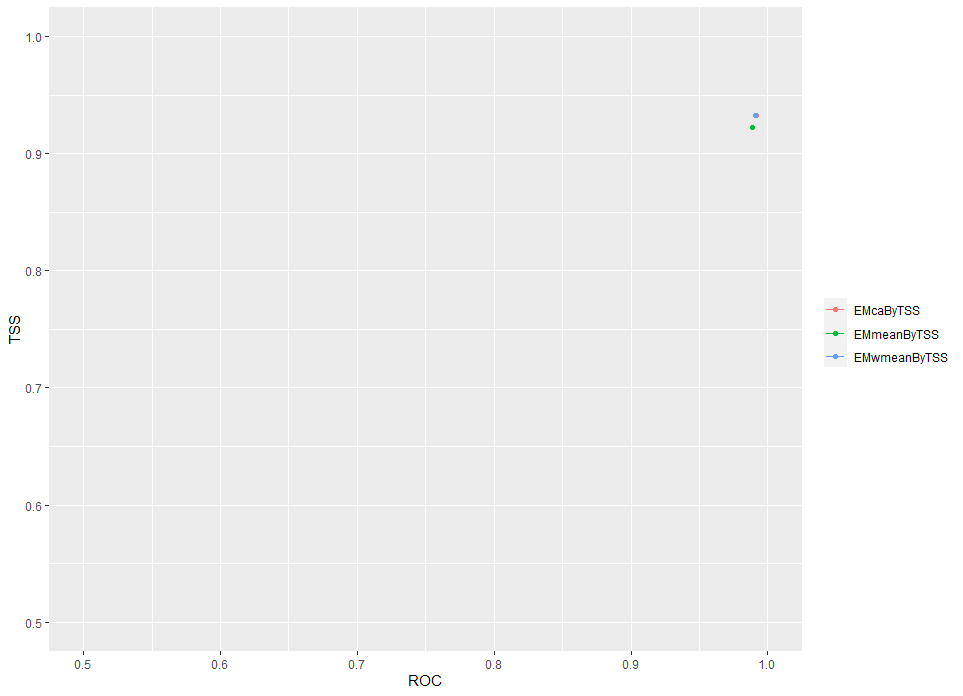


a

b

Fig. S3. Ensemble models’ evaluations of scores graph TSS versus ROC metrics to evaluate the probability of mean of ensemble model, probability of committee averaging, and probability of the mean weight for (a) R. aegypticus and (b) E. labiatus using different algorithms.

Table S2. Averaged variables importance for different models of R.aegypticus and E.labiatus habitat suitability and distribution

| Variables | *R.aegypticus* | | | | Average | *E.labiatus* | | | | Average |
| --- | --- | --- | --- | --- | --- | --- | --- | --- | --- | --- |
|  | GLM | GBM | RF | MaxEnt |  | GLM | GBM | RF | MaxEnt |  |
| Biol7 | 0.23975 | 0.07525 | 0.05825 | 0.03425 | 10.1875 | 0.06050 | 0.03950 | 0.04625 | 0.12250 | 6.71875 |
| Bio9 | 0.22300 | 0.02850 | 0.12825 | 0.06525 | 11.125 | 0.14125 | 0.00775 | 0.06900 | 0.07425 | 7.30625 |
| Bio14 | 0.06400 | 0.02375 | 0.06525 | 0.03050 | 4.5875 | 0.18800 | 0.03025 | 0.05475 | 0.25125 | 13.10625 |
| Bio15 | 0.48075 | 0.13325 | 0.07000 | 0.25500 | 23.475 | 0.44375 | 0.14175 | 0.08700 | 0.39800 | 26.7625 |
| Biol18 | 0.07575 | 0.04500 | 0.09675 | 0.04525 | 6.56875 | 0.09250 | 0.03975 | 0.08725 | 0.04075 | 6.50625 |
| Bio19 | 0.11300 | 0.05725 | 0.05350 | 0.09100 | 7.86875 | 0.08975 | 0.09425 | 0.03675 | 0.35250 | 14.33125 |
| Asp | 0.09325 | 0.03900 | 0.02100 | 0.01125 | 4.1125 | 0.03650 | 0.00475 | 0.01575 | 0.01175 | 1.71875 |
| DistWA | 0.20525 | 0.00700 | 0.02175 | 0.13475 | 9.21875 | 0.02175 | 0.00100 | 0.00275 | 0.00150 | 0.675 |
| DistWL | 0.07525 | 0.00025 | 0.00025 | 0.06600 | 3.54375 | 0.16425 | 0.00175 | 0.00150 | 0.01275 | 4.50625 |
| PonI | 0.10800 | 0.34950 | 0.28900 | 0.16375 | 22.75625 | 0.14775 | 0.42775 | 0.26450 | 0.53000 | 34.25 |
| LuLc | 0.23450 | 0.25925 | 0.09975 | 0.21000 | 20.0875 | 0.37400 | 0.19875 | 0.08425 | 0.20000 | 21.425 |
| Ecoregen | 0.29250 | 0.03225 | 0.04075 | 0.20625 | 14.29375 | 0.29150 | 0.01550 | 0.04100 | 0.14500 | 12.325 |
| Slope | 0.12850 | 0.04375 | 0.01350 | 0.00975 | 4.8875 | 0.02025 | 0.00150 | 0.00525 | 0.19000 | 5.425 |
| Veget | 0.24175 | 0.15250 | 0.17175 | 0.11100 | 16.925 | 0.32425 | 0.07725 | 0.18725 | 0.13200 | 18.01875 |

Table S3. Species range size change in each scenario ensemble using the committee averaging across predictions (ca) and the weighted sum of probabilities (wm). ‘ca’ committee averaging threshold, ‘wm’ weighted mean threshold for ensemble modeling of four algorithms.

| Time | | loss | unsuitable | remain suitable | gain | Perc-loss | Perc- gain | species  range change | Current range size | Future range size. nodisp | Future rangesize.fulldisp |  |
| --- | --- | --- | --- | --- | --- | --- | --- | --- | --- | --- | --- | --- |
| Current_2050_HG45 | | ca | 19600 | 1216791 | 55122 | 30571 | 26.23 | 40.91 | 14.682 | 74722 | 55122 | 85693 |
|  |  | wm | 19630 | 1213513 | 45814 | 43127 | 29.99 | 65.89 | 35.90 | 65444 | 45814 | 88941 |
|  |  | Ca* | 30027 | 1182535 | 87389 | 22048 | 25.573 | 18.78 | -6.79 | 117416 | 87389 | 109437 |
|  |  | wm | 16630 | 1220266 | 64038 | 21065 | 20.62 | 26.11 | 5.58 | 80668 | 64038 | 85103 |
| Current_2050_HG85 | | ca | 22618 | 1215093 | 52104 | 32269 | 30.27 | 43.19 | 12.92 | 74722 | 52104 | 84373 |
|  |  | wm | 20021 | 1201011 | 45423 | 55629 | 30.59 | 85.00 | 54.41 | 65444 | 45423 | 101052 |
|  |  | Ca* | 33590 | 1179404 | 83826 | 25179 | 28.61 | 21.44 | -7.16 | 117416 | 83826 | 109005 |
|  |  | wm | 18327 | 1215467 | 62341 | 25864 | 22.72 | 32.12 | 9.343 | 80668 | 62341 | 88205 |
| Current_2070_HG45 | | ca | 20150 | 1217565 | 54572 | 29797 | 26.97 | 39.88 | 12.91 | 74722 | 54572 | 84369 |
|  |  | wm | 19991 | 1213938 | 45453 | 42702 | 30.55 | 65.25 | 34.70 | 65444 | 45453 | 88155 |
|  |  | Ca | 30029 | 1182614 | 87387 | 21969 | 25.58 | 18.71 | -6.864 | 117416 | 87387 | 109356 |
|  |  | wm | 16662 | 1220372 | 64006 | 20959 | 20.66 | 25.98 | 5.33 | 80668 | 64006 | 84965 |
| Current_2070_HG85 | | Ca* | 27510 | 1215196 | 47212 | 32166 | 36.82 | 43.05 | 6.23 | 74722 | 47212 | 79378 |
|  |  | wm | 21764 | 1197573 | 43680 | 59067 | 33.26 | 90.26 | 57.00 | 65444 | 43680 | 102747 |
|  |  | Ca* | 40400 | 1177656 | 77016 | 26927 | 34.41 | 22.93 | -11.49 | 117416 | 77016 | 103943 |
|  |  | wm | 20900 | 1212136 | 59768 | 29195 | 25.91 | 36.19 | 10.28 | 80668 | 59768 | 88963 |

* *E. labiatus*


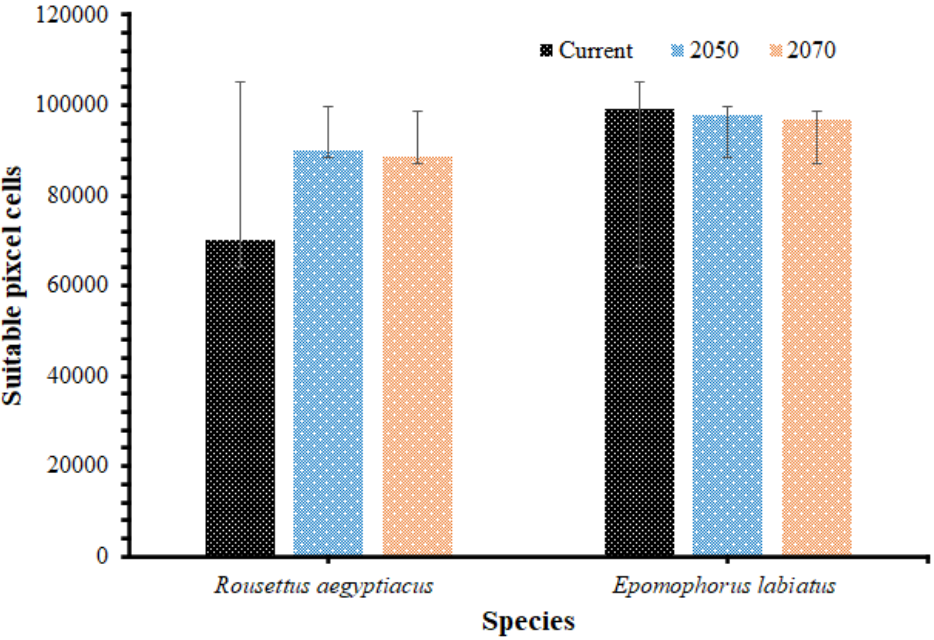


Figure. S4 The committee average and weighted mean prediction were used to calculate the ensemble predicted area of current and future suitable habitat predictions for *R. aegypticus and E. labiatus.*

**Appendix I**


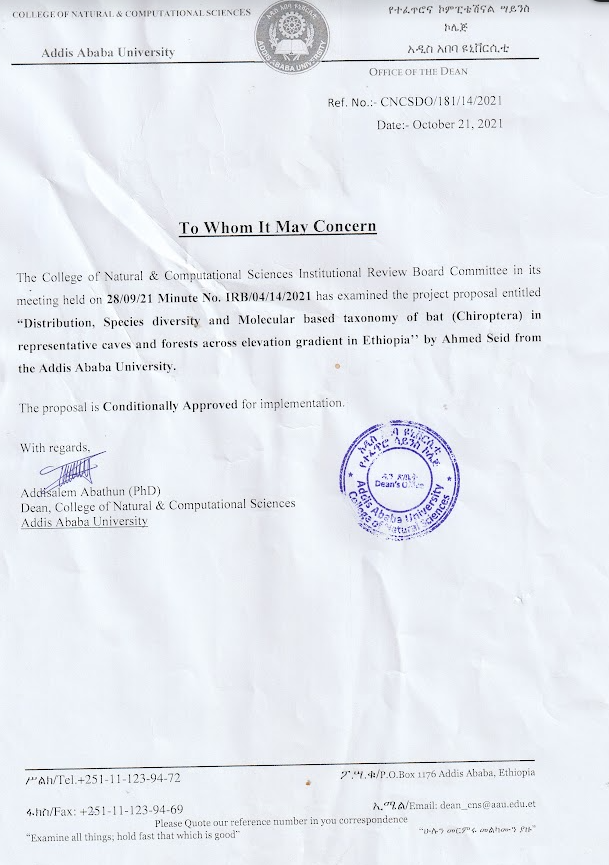


**Appendix 2**

**Biomod2 R software packages**

rm(list = ls())

## biomod2 video: Single species modelling ----

## download the required data ----

## setup environment ----

## load the required packages

library(recipes)

library(biomod2)

library(ggplot2)

library(gridExtra)

library(raster)

library(rasterVis)

## read data ----

setwd("C:\\Users\\user\\Desktop\\SDMRoussteus")

Rous_aegypticus_occ<-read.csv('R_aegp_occurence_point.csv')

summary(Rous_aegypticus_occ)

bioclim_curr<-

raster::stack(

c(Biol7 = "biol7.asc",

Bio9 = "Biol9.asc",

Bio14 = "Biol14.asc",

Bio15 = "Biol15.asc",

Biol18 = "Biol18.asc",

Bio19 = "Biol19.asc",

Asp = "Asp.asc",

DistWA = "DistWA.asc",

DistWL = "DistWL.asc",

PonI= "PopDIndex.asc",

LuLc = "LuLc.asc",

Ecoreg = "Ecoreg.asc",

Slope = "Slope.asc",

Veget = "veget.asc"))

# factorized the categorical variable

bioclim_curr$LuLc<-as.factor(bioclim_curr$LuLc)

bioclim_curr$Ecoreg<-as.factor(bioclim_curr$Ecoreg)

bioclim_curr$Veget<-as.factor(bioclim_curr$Veget)

bioclim_curr<-setMinMax(bioclim_curr)

bioclim_curr

# factorized the categorical variable

## format the data ----

RA_data<-

BIOMOD_FormatingData(

resp.var = Rous_aegypticus_occ['Rousettus.aegyptiacus'],

resp.xy = Rous_aegypticus_occ[, c('Longitude', 'Latitude')],

expl.var = bioclim_curr,

resp.name = "R.aegyptiacus",

eval.resp.var = NULL,

eval.expl.var = NULL,

eval.resp.xy = NULL,

PA.nb.rep =1,

PA.nb.absences = 10000,

PA.strategy = 'random',

PA.dist.min = NULL,

PA.dist.max = NULL,

PA.sre.quant = NULL,

PA.table = NULL,

na.rm = TRUE)

## formatted object summary

RA_data

## plot of selected pseudo-absences

plot(RA_data)

## define individual models options ----

#The aim of this function is to allow advanced user to change some default parameters of BIOMOD inner models. For each modeling technique, options can be set up.

#Each argument have to be put in a list object.

The best way to use this function is to print defaut models options (Print_Default_ModelingOptions) or create a default 'BIOMOD.model.option object' and print it in your console. Then copy the output, change only the required parameters, and paste it as function arguments. (see example).

## print default models options

#Print_Default_ModelingOptions()

DF_opt<-BIOMOD_ModelingOptions(

GLM = list(type = 'quadratic',

interaction.level = 0,

myFormula = NULL,

test = 'AIC',

family = binomial(link = 'logit'),

mustart = 0.5,

control = glm.control(epsilon = 1e-08, maxit = 50, trace = FALSE) ),

GBM = list( distribution = 'bernoulli',

n.trees = 2500,

interaction.depth = 7,

n.minobsinnode = 5,

shrinkage = 0.001,

bag.fraction = 0.5,

train.fraction = 1,

cv.folds = 3,

keep.data = FALSE,

verbose = FALSE,

perf.method = 'cv',

n.cores = 1),

GAM = list( algo = 'GAM_mgcv',

type = 's_smoother',

k = -1,

interaction.level = 0,

myFormula = NULL,

family = binomial(link = 'logit'),

method = 'GCV.Cp',

optimizer = c('outer','newton'),

select = FALSE,

knots = NULL,

paraPen = NULL,

control = list(nthreads = 1, irls.reg = 0, epsilon = 1e-07, maxit = 200, trace = FALSE, mgcv.tol = 1e-07

, mgcv.half = 15, rank.tol = 1.49011611938477e-08

, nlm = list(ndigit=7, gradtol=1e-06, stepmax=2, steptol=1e-04, iterlim=200, check.analyticals=0)

, optim = list(factr=1e+07), newton = list(conv.tol=1e-06, maxNstep=5, maxSstep=2, maxHalf=30, use.svd=0)

, outerPIsteps = 0, idLinksBases = TRUE, scalePenalty = TRUE, efs.lspmax = 15, efs.tol = 0.1, keepData = FALSE

, scale.est = NULL, edge.correct = FALSE) ),

CTA = list( method = 'class',

parms = 'default',

cost = NULL,

control = list(xval = 5, minbucket = 5, minsplit = 5, cp = 0.001, maxdepth = 25) ),

ANN = list( NbCV = 5,

size = NULL,

decay = NULL,

rang = 0.1,

maxit = 200),

SRE = list( quant = 0.025),

FDA = list( method = 'mars',

add_args = NULL),

MARS = list( type = 'simple',

interaction.level = 0,

myFormula = NULL,

nk = NULL,

penalty = 2,

thresh = 0.001,

nprune = NULL,

pmethod = 'backward'),

RF = list( do.classif = TRUE,

ntree = 500,

mtry = 'default',

nodesize = 5,

maxnodes = NULL),

MAXENT.Phillips = list( path_to_maxent.jar = 'C:/Users/user/Desktop/SDMRoussteus',

memory_allocated = 1024,

background_data_dir = 'default',

maximumbackground = 'default',

maximumiterations = 5000,

visible = FALSE,

linear = TRUE,

quadratic = TRUE,

product = TRUE,

threshold = TRUE,

hinge = TRUE,

lq2lqptthreshold = 80,

l2lqthreshold = 10,

hingethreshold = 15,

beta_threshold = -1,

beta_categorical = -1,

beta_lqp = -1,

beta_hinge = -1,

betamultiplier = 1,

defaultprevalence = 0.5))

#MAXENT.Phillips.2 =

# list(myFormula = NULL,

#regmult = 1,

# regfun = <function>))

#RA_opt <-

#BIOMOD_ModelingOptions(

# GLM = list(type = 'quadratic', interaction.level = 0),

# GBM = list(n.trees = 2500),

# GAM = NULL,

# CTA = NULL,

# ANN = NULL,

# SRE = NULL,

# FDA = NULL,

# MARS = NULL,

#RF = list(n.trees = 500),

# MAXENT.Phillips = list(maximumiterations = 1000,threshold=10,maximumbackground=10000))

## run the individual models ----

RA_models<-

BIOMOD_Modeling(

data = RA_data,

models = c("GLM", "GBM", "RF", "MAXENT.Phillips"),

models.options = DF_opt,

NbRunEval = 3,

DataSplit = 70,

Yweights = NULL,

Prevalence = NULL,

VarImport = 5,

models.eval.meth = c("KAPPA", "TSS", "ROC"),

SaveObj = TRUE,

rescal.all.models = FALSE,

do.full.models = TRUE,

modeling.id = "demo1" )

## asses individual models quality ----

## get models evaluation scores

RA_models_scores <- get_evaluations(RA_models)

RA_models_scores

## MF_models_scores is a 5 dimension array containing the scores of the models

dim(RA_models_scores)

dimnames(RA_models_scores)

## plot models evaluation scores

models_scores_graph(

RA_models,

by = "models",

metrics = c("ROC","TSS"),

xlim = c(0.5,1),

ylim = c(0.5,1))

models_scores_graph(

RA_models,

by = "cv_run" ,

metrics = c("ROC","TSS"),

xlim = c(0.5,1),

ylim = c(0.5,1))

models_scores_graph(

RA_models,

by = "data_set",

metrics = c("ROC","TSS"),

xlim = c(0.5,1),

ylim = c(0.5,1))

## check variable importance

(RA_models_var_import <- get_variables_importance(RA_models))

## make the mean of variable importance by algorithm

apply(RA_models_var_import, c(1,2), mean)

## individual models response plots

RA_glm <- BIOMOD_LoadModels(RA_models, models='GLM')

RA_gbm <- BIOMOD_LoadModels(RA_models, models='GBM')

RA_rf <- BIOMOD_LoadModels(RA_models, models='RF')

RA_Maxent<-BIOMOD_LoadModels(RA_models, models="MAXENT.Phillips")

glm_eval_strip <-

biomod2::response.plot2(

models = RA_glm,

Data = get_formal_data(RA_models,'expl.var'),

show.variables= get_formal_data(RA_models,'expl.var.names'),

col=c("blue","red","black","green"),

do.bivariate = FALSE,

fixed.var.metric = 'median',

legend = TRUE,

display_title = TRUE,

data_species = get_formal_data(RA_models,'resp.var') )

##Add a legend to it

#glm_eval_strip+legend("bottomright", legend=c("R.aegyptiacus_PA1_RUNG2_GLM","R.aegyptiacus_PA1_Full_GLM","R.aegyptiacus_PA1_Full_GLM","R.aegyptiacus_PA2_RUN1_GLM","R.aegyptiacus_PA2_RUN2_GLM"),

# pch=c(1,1),col=c(1,2,3,4),lty=c(1,1),cex=1.5,bty="n",ncol=1)

gbm_eval_strip <-

biomod2::response.plot2(

models = RA_gbm,

Data = get_formal_data(RA_models,'expl.var'),

show.variables= get_formal_data(RA_models,'expl.var.names'),

col=c("blue","red","black","green"),

do.bivariate = FALSE,

fixed.var.metric = 'median',

legend = TRUE,

display_title = TRUE,

data_species = get_formal_data(RA_models,'resp.var'))

##Add a legend to it

legend("botomright", legend=c("R.aegyptiacus_PA1_RUNG2_GLM","R.aegyptiacus_PA1_Full_GLM","R.aegyptiacus_PA1_Full_GLM","R.aegyptiacus_PA2_RUN1_GLM","R.aegyptiacus_PA2_RUN2_GLM"),

pch=c(1,1),col=c(1,2),lty=c(1,1),cex=1.5,bty="n",ncol=1)

rf_eval_strip <-

biomod2::response.plot2(

models = RA_rf,

Data = get_formal_data(RA_models,'expl.var'),

show.variables= get_formal_data(RA_models,'expl.var.names'),

col=c("blue","red","black","green"),

do.bivariate = FALSE,

fixed.var.metric = 'median',

legend = TRUE,

display_title = TRUE,

data_species = get_formal_data(RA_models,'resp.var'))

ANN_eval_strip <-

biomod2::response.plot2(

models = RA_ANN,

Data = get_formal_data(RA_models,'expl.var'),

show.variables= get_formal_data(RA_models,'expl.var.names'),

col=c("blue","red","black","green"),

do.bivariate = FALSE,

fixed.var.metric = 'median',

legend = TRUE,

display_title = TRUE,

data_species = get_formal_data(RA_models,'resp.var'))

MARS_eval_strip <-

biomod2::response.plot2(

models = RA_MARS,

Data = get_formal_data(RA_models,'expl.var'),

show.variables= get_formal_data(RA_models,'expl.var.names'),

col=c("blue","red","black","green"),

do.bivariate = FALSE,

fixed.var.metric = 'median',

legend = TRUE,

display_title = TRUE,

data_species = get_formal_data(RA_models,'resp.var'))

# gam_eval_strip <- biomod2::response.plot2(

# models = MF_gam,

# Data = get_formal_data(MF_models,'expl.var'),

#show.variables= get_formal_data(MF_models,'expl.var.names'),

#do.bivariate = FALSE,

#fixed.var.metric = 'median',

#legend = FALSE,

#display_title = FALSE,

#data_species = get_formal_data(MF_models,'resp.var'))

mx_eval_strip <-

biomod2::response.plot2(

models = RA_Maxent,

Data = get_formal_data(RA_models,'expl.var'),

show.variables= get_formal_data(RA_models,'expl.var.names'),

col=c("blue","red","black","green"),

do.bivariate = FALSE,

fixed.var.metric = 'median',

legend = TRUE,

display_title = TRUE,

data_species= get_formal_data(RA_models,'resp.var'))

## run the ensemble models ----

RA_ensemble_models <-

BIOMOD_EnsembleModeling(

modeling.output = RA_models,

em.by = 'all',

eval.metric = 'TSS',

eval.metric.quality.threshold = 0.7,

models.eval.meth = c('KAPPA','TSS','ROC'),

prob.mean = TRUE,

prob.cv = FALSE,

committee.averaging = TRUE,

prob.mean.weight = TRUE,

VarImport = 3)

## asses ensemble models quality ----

(RA_ensemble_models_scores <- get_evaluations(RA_ensemble_models))

## plot models evaluation scores

models_scores_graph(

RA_ensemble_models,

by = "models",

metrics = c("ROC","TSS"),

xlim = c(0.5,1),

ylim = c(0.5,1))

## do models projections ----

## current projections

RA_models_proj_current <-

BIOMOD_Projection(

modeling.output = RA_models,

new.env = bioclim_curr,

proj.name = "current",

binary.meth = "TSS",

output.format = ".img",

do.stack = FALSE,

build.clamping.mask = FALSE)

plot(RA_models_proj_current)

RA_ensemble_models_proj_current <-

BIOMOD_EnsembleForecasting(

EM.output = RA_ensemble_models,

projection.output = RA_models_proj_current,

binary.meth = "TSS",

output.format = ".img",

do.stack = FALSE)

plot(RA_ensemble_models_proj_current)

#setwd("C:/Users/user/Desktop/Bioodlab")

#Rous_aegyptiacus_occ<- read.csv('Occurence point.csv')

#summary(Rous_aegyptiacus_occ)

## future projections

## load 2050 bioclim variables for 4.5

bioclim_2050_HG45<-

stack(

c(Biol7 = "HadGEM2050_45_Bio7.asc",

Bio9 = "HadGEM2050_45_Bio9.asc",

Bio14= "HadGEM2050_45_Bio14.asc",

Bio15= "HadGEM2050_45_Bio15.asc",

Biol18 = "HadGEM2050_45_Bio18.asc",

Bio19 = "HadGEM2050_45_Bio19.asc",

Asp = "Asp.asc",

DistWA = "DistWA.asc",

DistWL = "DistWL.asc",

PonI= "PopDIndex.asc",

LuLc = "LuLc.asc",

Ecoreg = "Ecoreg.asc",

Slope = "Slope.asc",

Veget = "veget.asc"))

RA_models_proj_2050_HG45 <-

BIOMOD_Projection(

modeling.output = RA_models,

new.env = bioclim_2050_HG45,

proj.name = "2050_HG45",

binary.meth = "TSS",

output.format = ".img",

do.stack = FALSE,

build.clamping.mask = FALSE)

RA_ensemble_models_proj_2050_HG45 <-

BIOMOD_EnsembleForecasting(

EM.output = RA_ensemble_models,

projection.output = RA_models_proj_2050_HG45,

binary.meth = "TSS",

output.format = ".img",

do.stack = FALSE)

plot(RA_ensemble_models_proj_2050_HG45, str.grep = "EMca|EMwmean")

## load 2070 bioclim variables 4.5

bioclim_2070_HG45<-

stack(

c(

Biol7 = "HadGEM2070_45_Bio7.asc",

Bio9 = "HadGEM2070_45_Bio9.asc",

Bio14 = "HadGEM2070_45_Bio14.asc",

Bio15 = "HadGEM2070_45_Bio15.asc",

Biol18= "HadGEM2070_45_Bio18.asc",

Bio19 = "HadGEM2070_45_Bio19.asc",

Asp = "Asp.asc",

DistWA = "DistWA.asc",

DistWL = "DistWL.asc",

PonI= "PopDIndex.asc",

LuLc = "LuLc.asc",

Ecoreg = "Ecoreg.asc",

Slope = "Slope.asc",

Veget = "veget.asc" ))

RA_models_proj_2070_HG45<-

BIOMOD_Projection(

modeling.output = RA_models,

new.env = bioclim_2050_HG45,

proj.name = "2070_BC45",

binary.meth = "TSS",

output.format = ".img",

do.stack = FALSE,

build.clamping.mask=FALSE)

RA_ensemble_proj_2070_HG45<-

BIOMOD_EnsembleForecasting(

EM.output = RA_ensemble_models,

projection.output = RA_models_proj_2070_HG45,

binary.meth = "TSS",

output.format = ".img",

do.stack = FALSE

)

## check how projections looks like

plot(RA_ensemble_proj_2070_HG45, str.grep = "EMca|EMwmean")

# load 2050 bioclimate variables 8.5

bioclim_2050_HG85<-

stack(

c( Biol7 = "HadGEM2050_85_Bio7.asc",

Bio9 = "HadGEM2050_85_Bio9.asc",

Bio14 = "HadGEM2050_85_Bio14.asc",

Bio15 = "HadGEM2050_85_Bio15.asc",

Biol18 = "HadGEM2050_85_Bio18.asc",

Bio19 = "HadGEM2050_85_Bio19.asc",

Asp = "Asp.asc",

DistWA = "DistWA.asc",

DistWL = "DistWL.asc",

PonI= "PopDIndex.asc",

LuLc = "LuLc.asc",

Ecoreg = "Ecoreg.asc",

Slope = "Slope.asc",

Veget = "veget.asc")

)

RA_models_proj_2050_HG85 <-

BIOMOD_Projection(

modeling.output = RA_models,

new.env = bioclim_2050_HG85,

proj.name = "2050_HG85",

binary.meth = "TSS",

output.format = ".img",

do.stack = FALSE,

build.clamping.mask=FALSE

)

RA_ensemble_models_proj_2050_HG85 <-

BIOMOD_EnsembleForecasting(

EM.output = RA_ensemble_models,

projection.output = RA_models_proj_2050_HG85,

binary.meth = "TSS",

output.format = ".img",

do.stack = FALSE

)

plot(RA_ensemble_models_proj_2050_HG85, str.grep = "EMca|EMwmean")

# load 2070 bio climate variables for scenarios 8.5

bioclim_2070_HG85 <-

stack(

c(Biol7 = "HadGEM2070_85_Bio7.asc",

Bio9 = "HadGEM2070_85_Bio9.asc",

Bio14 = "HadGEM2070_85_Bio14.asc",

Bio15 = "HadGEM2070_85_Bio15.asc",

Biol18 = "HadGEM2070_85_Bio18.asc",

Bio19 = "HadGEM2070_85_Bio19.asc",

Asp = "Asp.asc",

DistWA = "DistWA.asc",

DistWL = "DistWL.asc",

PonI= "PopDIndex.asc",

LuLc = "LuLc.asc",

Ecoreg = "Ecoreg.asc",

Slope = "Slope.asc",

Veget = "veget.asc")

)

RA_models_proj_2070_HG85 <-

BIOMOD_Projection(

modeling.output = RA_models,

new.env = bioclim_2070_HG85,

proj.name = "2070_HG85",

binary.meth = "TSS",

output.format = ".img",

do.stack = FALSE,

build.clamping.mask=FALSE

)

RA_ensemble_models_proj_2070_HG85 <-

BIOMOD_EnsembleForecasting(

EM.output = RA_ensemble_models,

projection.output = RA_models_proj_2070_HG85,

binary.meth = "TSS",

output.format = ".img",

do.stack = FALSE

)

plot(RA_ensemble_models_proj_2070_HG85, str.grep = "EMca|EMwmean")

## compute Species Range Change (SRC) ----

## load binary projections

RA_bin_proj_current <-

stack( c(ca = "R.aegyptiacus/proj_current/individual_projections/R.aegyptiacus_EMcaByTSS_mergedAlgo_mergedRun_mergedData_TSSbin.img",

wm = "R.aegyptiacus/proj_current/individual_projections/R.aegyptiacus_EMwmeanByTSS_mergedAlgo_mergedRun_mergedData_TSSbin.img"

) )

RA_bin_proj_2050_HG45 <-

stack(c(ca = "R.aegyptiacus/proj_2050_HG45/individual_projections/R.aegyptiacus_EMcaByTSS_mergedAlgo_mergedRun_mergedData_TSSbin.img",

wm = "R.aegyptiacus/proj_2050_HG45/individual_projections/R.aegyptiacus_EMwmeanByTSS_mergedAlgo_mergedRun_mergedData_TSSbin.img"

) )

RA_bin_proj_2070_HG45 <-

stack(c(ca = "R.aegyptiacus/proj_2070_BC45/individual_projections/R.aegyptiacus_EMcaByTSS_mergedAlgo_mergedRun_mergedData_TSSbin.img",

wm = "R.aegyptiacus/proj_2070_BC45/individual_projections/R.aegyptiacus_EMwmeanByTSS_mergedAlgo_mergedRun_mergedData_TSSbin.img"

) )

RA_bin_proj_2050_HG85 <-

stack(c(ca = "R.aegyptiacus/proj_2050_HG85/individual_projections/R.aegyptiacus_EMcaByTSS_mergedAlgo_mergedRun_mergedData_TSSbin.img",

wm = "R.aegyptiacus/proj_2050_HG85/individual_projections/R.aegyptiacus_EMwmeanByTSS_mergedAlgo_mergedRun_mergedData_TSSbin.img"

) )

RA_bin_proj_2070_HG85 <-

stack(c(ca = "R.aegyptiacus/proj_2070_HG85/individual_projections/R.aegyptiacus_EMcaByTSS_mergedAlgo_mergedRun_mergedData_TSSbin.img",

wm = "R.aegyptiacus/proj_2070_HG85/individual_projections/R.aegyptiacus_EMwmeanByTSS_mergedAlgo_mergedRun_mergedData_TSSbin.img"

) )

## SRC current -> 2050-HG45

SRC_current_2050_HG45 <-

BIOMOD_RangeSize(

RA_bin_proj_current,

RA_bin_proj_2050_HG45)

SRC_current_2050_HG45$Compt.By.Models

## SRC current -> 2050-HG85

SRC_current_2050_HG85 <-

BIOMOD_RangeSize(

RA_bin_proj_current,

RA_bin_proj_2050_HG85)

SRC_current_2050_HG85$Compt.By.Models

## SRC current -> 2070-45

SRC_current_2070_HG45 <-

BIOMOD_RangeSize(

RA_bin_proj_current,

RA_bin_proj_2070_HG45)

SRC_current_2070_HG45$Compt.By.Models

## SRC current -> 2070-85

SRC_current_2070_HG85 <-

BIOMOD_RangeSize(

RA_bin_proj_current,

RA_bin_proj_2070_HG85)

SRC_current_2070_HG85$Compt.By.Models

# Stack the current and future scenarios to plot the src with two path (curr-2050_45-2070_45 and curr_2050_85-2070_85)

RA_src_map_2050 <-

stack(

SRC_current_2050_HG45$Diff.By.Pixel,

SRC_current_2070_HG45$Diff.By.Pixel)

RA_src_map_2070 <-

stack(

SRC_current_2050_HG85$Diff.By.Pixel,

SRC_current_2070_HG85$Diff.By.Pixel)

names(RA_src_map_2050) <- c("ca cur-2050-HG45", "wm cur-2050-HG45", "ca cur-2070-HG45", "wm cur-2070-HG45")

names(RA_src_map_2070) <- c("ca cur-2050-HG85", "wm cur-2050-HG85", "ca cur-2070-HG85", "wm cur-2070-HG85")

my.at <- seq(-2.5, 1.5, 1)

myColorkey <-

list(

at = my.at, ## where the colors change

labels =

list(

labels = c("lost", "pres", "abs","gain"), ## labels

at = my.at[-1] - 0.5 ## where to print labels

))

rasterVis::levelplot(

RA_src_map_2050,

main = "R.aegyptiacus range change",

colorkey = myColorkey,

col.regions=c('#f03b20', '#99d8c9', '#f0f0f0', '#2ca25f'),

layout = c(2,2))

rasterVis::levelplot(

RA_src_map_2070,

main = "R.aegyptiacus range change",

colorkey = myColorkey,

col.regions=c('#f03b20', '#99d8c9', '#f0f0f0', '#2ca25f'),

layout = c(2,2))

## compute the stratified density of probabilities on SRC ----

## the reference projection

ref_ca <- subset(RA_bin_proj_current, "ca")

ref_wm <- subset(RA_bin_proj_current, "wm")

## define the facets we want to study

mods <- c("GLM","GBM","RF", "MAXENT.Phillips")

data_set <- c("PA1")

cv_run <- c("RUN1", "RUN2","RUN3", "Full")

## construct combination of all facets

groups <-

as.matrix(

expand.grid(

models = mods,

data_set = data_set,

cv_run = cv_run,

stringsAsFactors = FALSE))

## load all projections we have produced

all_bin_proj_files <-

list.files(

path = "R.aegyptiacus",

pattern = "_TSSbin.img$",

full.names = TRUE,

recursive = TRUE)

### current versus 2050 (removed the projections for current and 2070)

current_and_2050_HG45_proj_files <- grep(all_bin_proj_files, pattern="2050_HG45", value=T)

current_and_2050_HG85_proj_files <- grep(all_bin_proj_files, pattern="2050_HG85", value=T)

current_and_2070_HG45_proj_files <- grep(all_bin_proj_files, pattern="2070_BC45", value=T)

current_and_2070_HG85_proj_files <- grep(all_bin_proj_files, pattern="2070_HG85", value=T)

## keep only projections that match with our selected facets groups

selected_bin_proj_2050_HG45_files <-

apply(

groups, 1,

function(x){

proj_file <- NA

match_tab <- sapply(x, grepl, current_and_2050_HG45_proj_files)

match_id <- which(apply(match_tab, 1, all))

if(length(match_id)) proj_file <- current_and_2050_HG45_proj_files[match_id]

proj_file})

selected_bin_proj_2050_HG85_files <-

apply(

groups, 1,

function(x){

proj_file <- NA

match_tab <- sapply(x, grepl, current_and_2050_HG85_proj_files)

match_id <- which(apply(match_tab, 1, all))

if(length(match_id)) proj_file <- current_and_2050_HG85_proj_files[match_id]

proj_file})

selected_bin_proj_2070_HG45_files <-

apply(

groups, 1,

function(x){

proj_file <- NA

match_tab <- sapply(x, grepl, current_and_2070_HG45_proj_files)

match_id <- which(apply(match_tab, 1, all))

if(length(match_id)) proj_file <- current_and_2070_HG45_proj_files[match_id]

proj_file})

selected_bin_proj_2070_HG85_files <-

apply(

groups, 1,

function(x){

proj_file <- NA

match_tab <- sapply(x, grepl, current_and_2070_HG85_proj_files)

match_id <- which(apply(match_tab, 1, all))

if(length(match_id)) proj_file <- current_and_2070_HG85_proj_files[match_id]

proj_file})

## remove no-matching groups

to_remove_2050_45 <- which(is.na(selected_bin_proj_2050_HG45_files))

if(length(to_remove_2050_45)){

groups <- groups[-to_remove_2050_45,]

selected_bin_proj_2050_HG45_files <- selected_bin_proj_2050_HG45_files[-to_remove_2050_45]}

to_remove_2050_85 <- which(is.na(selected_bin_proj_2050_HG85_files))

if(length(to_remove_2050_85)){

groups <- groups[-to_remove_2050_85 ,]

selected_bin_proj_2050_HG85_files <- selected_bin_proj_2050_HG85_files[-to_remove_2050_85]}

to_remove_2070_45 <- which(is.na(selected_bin_proj_2070_HG45_files))

if(length(to_remove_2070_45)){

groups <- groups[-to_remove_2070_45,]

selected_bin_proj_2070_HG45_files <- selected_bin_proj_2070_HG45_files[-to_remove_2070_45]}

to_remove_2070_85 <- which(is.na(selected_bin_proj_2070_HG85_files))

if(length(to_remove_2070_85)){

groups <- groups[-to_remove_2070_85,]

selected_bin_proj_2070_HG85_files<- selected_bin_proj_2070_HG85_files[-to_remove_2070_85]}

## build stack of selected projections

proj_groups_2050_45 <- stack(selected_bin_proj_2050_HG45_files)

proj_groups_2050_85 <- stack(selected_bin_proj_2050_HG85_files)

proj_groups_2070_45 <- stack(selected_bin_proj_2070_HG45_files)

proj_groups_2070_85 <- stack(selected_bin_proj_2070_HG85_files)

# plot probability density functions

ProbDensFunc(

initial = as.vector(ref_ca),

projections = raster::as.matrix(proj_groups_2050_45 ),

groups = t(groups),

plothist = TRUE,

resolution = 10,

cvsn = TRUE)

ProbDensFunc(

initial = as.vector(ref_ca),

projections = raster::as.matrix(proj_groups_2050_85),

groups = t(groups),

plothist = TRUE,

resolution = 10,

cvsn = TRUE)

ProbDensFunc(

initial = as.vector(ref_ca),

projections = raster::as.matrix(proj_groups_2070_45),

groups = t(groups),

plothist = TRUE,

resolution = 10,

cvsn = TRUE)

ProbDensFunc(

initial = as.vector(ref_ca),

projections = raster::as.matrix(proj_groups_2070_85),

groups = t(groups),

plothist = TRUE,

resolution = 10,

cvsn = TRUE)

# OR if you need the weighted mean probabilities use the following

ProbDensFunc(

initial = as.vector(ref_wm),

projections = raster::as.matrix(proj_groups_2050_45),

groups = t(groups),

plothist = TRUE,

resolution = 10,

cvsn = TRUE)

ProbDensFunc(

initial = as.vector(ref_wm),

projections = raster::as.matrix(proj_groups_2050_85),

groups = t(groups),

plothist = TRUE,

resolution = 10,

cvsn = TRUE)

ProbDensFunc(

initial = as.vector(ref_wm),

projections = raster::as.matrix(proj_groups_2070_45),

groups = t(groups),

plothist = TRUE,

resolution = 10,

cvsn = TRUE)

ProbDensFunc(

initial = as.vector(ref_wm),

projections = raster::as.matrix(proj_groups_2070_85),

groups = t(groups),

plothist = TRUE,

resolution = 10,

cvsn = TRUE)
